# Go With Your Gut: The Shaping of T-Cell Response by Gut Microbiota in Allergic Asthma

**DOI:** 10.3389/fimmu.2020.01485

**Published:** 2020-07-14

**Authors:** Alessandro Di Gangi, Maria Elisa Di Cicco, Pasquale Comberiati, Diego G. Peroni

**Affiliations:** ^1^Department of Clinical and Experimental Medicine, Section of Pediatrics, University of Pisa, Pisa, Italy; ^2^Department of Clinical Immunology and Allergology, I.M. Sechenov First Moscow State Medical University, Moscow, Russia

**Keywords:** gut microbiota, asthma, children, T lymphocytes, microbiome

## Abstract

Novel methods in immunological research and microbiome evaluation have dramatically changed several paradigms associated with the pathogenesis of allergic asthma (AAS). Ovalbumin and house dust mite-induced AAS in germ-free or specific pathogen-free mice are the two leading experimental platforms that significantly contribute to elucidate the relationship between AAS and gut microbiota. Beyond the exacerbation of T helper (Th) 2 responses, a complex network of immunological interaction driven by gut microbiota could modulate the final effector phase. Regulatory T cells are abundant in gastrointestinal mucosa and have been shown to be pivotal in AAS. The gut microbiota could also influence the activity of other T cell subsets such as Th9, Th17, and populations of effector/memory T lymphocytes. Furthermore, gut microbiota metabolites drive the hematopoietic pattern of dendritic cells and ameliorate lung Th2 immunity in AAS models. The administration of probiotics has shown conflicting results in AAS, and limited evidence is available on the immunological pathways beyond their activity. Moreover, the impact of early-life gut dysbiosis on AAS is well-known both experimentally and clinically, but discrepancies are observed between preclinical and clinical settings. Herein, our aim is to elucidate the most relevant preclinical and clinical scenarios to enlighten the potential role of the gut microbiota in modulating T lymphocytes activity in AAS.

## T Cell Plasticity and Gut Microbiota

During homeostatic periods, gut-microbiota and T-cells within the gut mucosa engaged profitable crosstalk capable of shaping the systemic immune response of the individual. T-cell-receptors are reactive to microbiota-derived antigens that are necessary for an adequate maturation of the immune system and to ensure proper colonization of the gut lumen. Heterogeneous microbiota-derived signals could drive the polarization of Th cells into four different categories: while Th1 (1) and Th17 (2) dysregulation are relevant in inflammatory and autoimmune diseases, GATA3^+^ Th2 responses (3) and T regulatory lymphocytes (Tregs) (4) are pivotal in allergic responses. Some bacteria metabolites, such as short-chain fatty acids (SCFAs), could regulate the tolerogenic effect of Tregs and they are directly sensed by G-protein-coupled-receptors (GPCRs) (5). Alternatively, Tregs polarization could be promoted by an IL-10 dependent pathway from microbiota-derived antigen presentation by dendritic cells (DCs) (6). Several other Th subsets such as Th9 (7) and Tregs subtypes (8) are implicated in allergic responses while their relationship with the gut microbiota is not entirely defined. Furthermore, the role of Th17 in AAS was heavily investigated (9) and they are currently recognized as one of the crucial mediators of AAS.

## What We Have Learned From Gut Microbiota Manipulation in Murine Models of Allergic Asthma

More than a decade ago, evidence in murine models rose in support of the hygiene hypothesis, when Th2 response was associated with a state of gut dysbiosis (10). Nowadays, the paradigm has evolved, and the complex immunological network beyond the gut-lung axis is under active research.

### The Role of *Clostridium spp* Emphasize Regulatory T-Cell Activity

A number of studies identified *Clostridium spp* as crucial modulators of AAS. First, insights come from the investigation on the impact of antibiotic therapies on T cell populations. It was demonstrated that CD4^+^CD25^+^FoxP3^+^ Tregs are slightly reduced when mice are treated with polymixin B or vancomycin but not streptomycin withing the intestinal wall, while lung Tregs frequencies are comparable among vancomycin or streptomycin-treated animals or controls (11, 12). Interestingly, the ovalbumin (OVA) challenge exacerbates AAS in vancomycin-treated animals but not within the streptomycin group (12). It is well-known that vancomycin preferentially targets *Clostridium spp*. In order to further elucidate the role of these strains in AAS, stool transfer experiments between mice were conducted. Murine models colonized with fecal water derived from mice supplemented with a mixture of *Clostridium* strains have demonstrated a higher percentage of IL10^+^CTLA-4^high^IKZF2^−^ colonic Tregs when compared to controls while a similar approach with *Lactobacillus spp* and *Bacteroides spp* failed to show any significant variation (11). Of note, in this context, Tregs does not express IKZF2, an essential transcription factor that is necessary for the stabilization of their suppressive activity in autoinflammatory models (13). A similar approach with fecal material from human volunteers has demonstrated that clusters of Clostridia IV/XIV and XVIII are able to increase the frequency of IL10^+^ICOS^+^CTLA4^+^CD25^+^ Tregs in colon mucosa and they are implicated in protection against OVA-induced Th2 colitis (14). However, their relevance in AAS is under debate. One of the significant limitations of these studies is represented by the absence of a detailed functional characterization of T-cells; for instance, IL-10 and CTLA-4 are essential for the immunosuppressive activity of Tregs, while ICOS is crucial for their effector activity rather than their induction (15) thus, the contribution of different T effector/memory (Tem) populations should be addressed.

### The Importance of Effector/Memory T Cells Revealed by *Bacteroides spp*

Observations in AAS models enhance the role of *Bacteroides spp*. Even if they are not a direct target of vancomycin, perinatal treatment in mice reduces *Bacteroides spp* and relatively increases *Lactobacillus spp* (16). Importantly, the effect of vancomycin on AAS is evident only during early-life and not during adulthood, identifying a specific “window of opportunity” (16). Mechanistic studies on *Bacteroides fragilis* in AAS provide further insights into the regulation of T cell response. In other settings, it was shown that the polysaccharide A (PSA) is a non-protein antigen presented by MHC-II (17) capable of activating CD4^+^ cells (18). The oral exposure to PSA protects murine models against AAS trough a T-cell mediated pathway (19). PSA usually elicits a FoxP3^+^ peripheral Tregs response in mice and FoxP3^−^ Tregs cells in humans (20). However, in mice primed with PSA and sensitized to OVA, the PSA-responding population is not composed of FoxP3^+^ Tregs but relies on IL-10 to exert its protective effect (19). Thus, it seems that PSA could protect against AAS through an IL-10 dependent mechanism, but the source of IL-10 was unclear. The adoptive transfer approach of PSA-responding CD4^+^ T lymphocytes between IL10^−/−^ and wild type animals allows the recognition that PSA-responding T cells are a population of FoxP3^−^CD45Rb^low^CD44^+^CD62L^−^ Tem that support the production of IL-10 by lung resident FoxP3^+^ Tregs (21). To date, it is not known if other gut-derived antigens could act indirectly on lungs T response in a similar way.

### Modulation of the Gut-Lung Axis Through Antibiotics and Probiotics Impacts the T-Cell Response

Mice treated with a combined intermittent antibiotic regimen early after weaning and subsequently challenged with house dust mite (HDM) show a significant reduction of the FoxP3^+^/CD4^+^ ratio in mediastinal lymph nodes (MLNs), that is proportional to the Simpson diversity index variation of the fecal microbiome (22). Therefore, a direct link between Tregs homeostasis and gut microbiota in AAS exists, and a state of dysbiosis could strongly influence the severity of Th2-mediated inflammation. Among the limitations of this approach, the use of aggressive and intensive antibiotic regimens is one of the most relevant obstacles for translation into clinical practice. Although direct evidence of its implication in AAS is currently lacking, other works address the long-lasting immunomodulatory effects of an early-life single macrolide course vs. a three pulses course (23). In this context, intestinal CD4^+^IL17A^+^ T lymphocytes are decreased while CD4^+^IFN-γ T cells are not affected, and CD4^+^FoxP3^+^ Tregs percentage is only slightly increased among treated animals (23). Of note, only slight differences are noted in germ-free mice, and the degree of systemic immune perturbance of a single macrolide pulse is relatively modest, but it is strong enough to induce an imbalance in local Th17 immunity, leading to long-lasting microbiome alterations (23). It would be of great interest to explore T-cell development in a similar model challenged with HDM or OVA.

Since transient gut dysbiosis is relatively common in children due to the widespread use of antibiotics (24), it is possible that an early probiotic administration could recover this state and drive the Th1/Th2 balance. Supplementation with *Lactobacillus Ramnosus* or *Bifidobacterium lactis* is proven to be an efficient AAS suppressor and a robust inductor of FoxP3^+^ Tregs in MLNs of newborn OVA-sensitized mice (25). Moreover, the proliferative response to probiotic administration enhances the production of TGF-β secreting CD4^+^/CD3^+^ T lymphocytes (25), thereby contributing to the establishment of a tolerogenic environment. Probiotic treatment has been shown to be effective in protecting newborn mice against AAS while it has been proven to be ineffective in adult mice. Interestingly, both adult and newborn mice are able to induce a CD4^+^FoxP3^+^ Treg response in the lungs after probiotic administration, but when splenocytes from tolerant wild-type mice were adoptively transferred into adult mice, only newborn's Tregs are able to control pulmonary inflammation (26). Therefore, the generation of Tregs itself is not sufficient to confer protection to AAS, but an intrinsic feature of neonatal Tregs is necessary (26), and it would be of great interest for future research.

### Diet Modulation of T Cell Response Is Only Partially Driven by Gut Microbiota Alterations

Environmental factors play a crucial role in the development of AAS, and specific maternal diets could be beneficial in preventing the onset of AAS. Bacteria metabolize fermentable dietary fiber into SCFAs, small soluble molecules that could trigger strong immunomodulatory effects (27). SCFAs diet content is capable of driving the gut microbiota composition, and this shift could lead to significant protection against AAS (5). Interestingly, vancomycin treatment in mice mainly delates bacteria capable of producing SCFAs (28).

**Figure 1:**
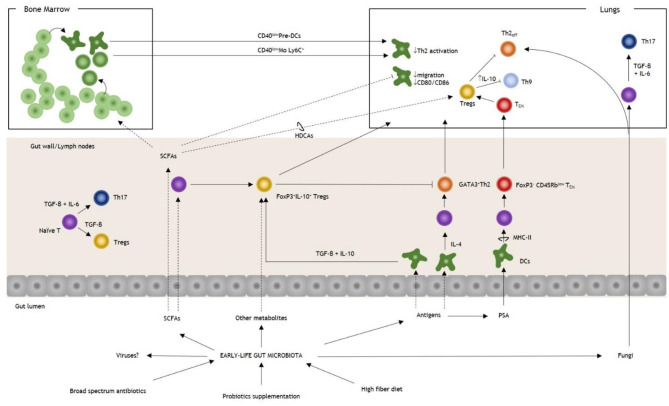
Overview of gut microbiota and T cell modulation in allergic asthma. Early-life perturbations, such as broad-spectrum antibiotics, diet, and probiotics, are essential in determining the risk of allergic asthma. Antigens derived from the gut microbiota stimulate the proliferation of DCs, and they could trigger both Th2 or Tregs responses under the influence of IL-4 or TGF-β+IL-10, respectively. PSA of *Bacteroides fragilis* stimulates the expansion of a FoxP3^−^ Tem population that regulates lung immunity through the induction of FoxP3^+^ Tregs. SCFAs modulate T cell response, both priming naïve T cells and upregulating the FoxP3 promoter in lung Tregs. SCFAs influence the hematopoietic pattern in a context-dependent way. In AAS, they increase the production of DCs and monocytes progenitors with the generation of pre-DCs and inflammatory Mo Ly6C^+^. During AAS inflammation, after their homing to the lungs, they express reduced levels of CD40 (CD40^low^ DCs and CD40^low^ Mo, respectively), impairing the action of Th2_eff_. Among SCFAs, butyrate could promote the induction of pulmonary Tregs trough HDCAs leading to the inhibition of Th9 activity and ameliorating AAS. Fungal dysbiosis could trigger both a Th2 and a Th17 response, thereby increasing the susceptibility to AAS. Note that under the influence of TGF-β+IL-6 T cells are polarized to Th17 while in the absence of IL-6 Tregs are induced. It is not known if intestinal viruses could have a direct effect on T-cell response in AAS. AAS, allergic asthma; DCs, dendritic cells; IL, interleukin; Mo, monocytes; PSA, polysaccharide antigen; SCFAs, small chain fatty acids; Tem, T effector memory; TGF-β, tumor growth-factor β; Th, T helper cell; Th2_eff_, T helper 2 effector; Treg, T regulatory cell.

After a high-fiber diet, the gut microbiota is enriched with *Bacteoidaceae* and *Bifidobacteriaceae*, while SCFAs, particularly acetate and propionate, increased (5). However, SCFAs are not detected in lung tissue after HDM challenges; thus, they exert their action through an indirect mechanism (5). Furthermore, butyrate enhances the extrathymic generation of Tregs (29), but a high-fiber diet was not associated with an increase in CD25^+^FoxP3^+^ Tregs (5). Therefore, other mechanisms should underline this effect. Bone-marrow derived DCs were investigated as possible metabolite-specific mediators. It was demonstrated that butyrate significantly alters DCs gene expression, reduces costimulatory molecules and impairs CCL19-dependent DCs migration (28). On the other hand, propionate acts via GPCRs in a context-dependent manner, enhancing the hematopoietic activity of DCs precursors that could impair Th2 activity in the lungs (Figure 1) (27). Therefore, gut microbiota diet perturbations could indirectly affect the Th2 response through the modulation of the hematopoietic activity in the bone marrow through a metabolite-specific pathway. In addition to this mechanism, SCFAs can establish an anti-inflammatory activity by the direct interaction with the histone deacetylase (HDAC) protein family (30). Of note, HDAC proteins confer a permissive chromatin structure that enhances the transcription of involved regions (27). Propionate, but not acetate, could inhibit HDACs, enhancing the extrathymic Treg generation promoted by butyrate (29). According to this finding, dramatic protection against AAS after HDM exposure in progeny was related to the downregulation of *Nppa* that inhibits HDAC-9 and increases the acetylation rates of the FoxP3 promoter (30). Furthermore, this action was reported as independent from the microbiota shift observed in treated mice (30).

Other T-cell subsets could be involved in SCFAs-mediated protection against AAS. In newborn mice treated with a commercially available mixture of probiotics, increased levels in butyrate, but not other metabolites, are able to induce the proliferation of Tregs and negatively modulate OVA-induced AAS (31). Since Tregs are potent inhibitors of Th9 (32), their capacity to reduce IL-9 expression and Th9 differentiation was assessed. Butyrate-treated mice show lower frequencies of Th9 cells in the lungs, while no difference is observed for Th2 cells (31). Moreover, adoptive transfer of Th9 or IL-9 administration could revert the protective effect of butyrate, indicating that the mechanisms are partially related to this Th subclass (31). Lastly, while the link between obesity and non-allergic bronchial hyperresponsiveness is widely accepted, a high-fat diet has little effect in modulating Tregs responses by an impairment of DCs activation in OVA-induced AAS (33). Therefore, diet could have a significant role during early developmental stages, and it is capable of shaping the T-cell effector phase through a variety of mechanisms.

### The Hidden World: Fungi and Viruses

Fungal dysbiosis was recently recognized as one of the foremost promoters of AAS in experimental models, but the mechanisms are only partially understood. Fluconazole can exacerbate AAS in mice and increase IL-4^+^CD4^+^ and IL-13^+^CD4^+^ T-cells (34). In another study, *Wallemia mellicola* colonizes the intestine efficiently after depletion of resident microbiota by wide-spectrum antibiotics and promotes the accumulation of IL-13^+^CD4^+^ T-cells in MLNs and AAS exacerbations (35). Therefore, the induction of Th2 responses seems to be cardinal during mycobiota dysbiosis. However, *C. albicans spp*. were able to increase AAS susceptibility through RORδT^+^ T-cells (36) and recent findings in mice treated with fluconazole and then “recovered” with oral gavage of three dysbiotic fungal species demonstrate that AAS susceptibility is mediated by a mixed GATA3^+^ and RORδT^+^ T-cell response (37). Therefore, both Th2 and Th17 are involved in AAS during mycobiota dysbiosis, but further studies are needed to enlighten the mechanisms and possible therapeutic opportunities. Of note, Th17 are induced under the presence of both IL-6 and TGF-β while the absence of IL-6 promotes the differentiation into Tregs (4). To our knowledge, there is a lack of evidence on mechanisms that could link intestinal viruses and T lymphocytes response in AAS; it would be of great interest to address this topic in the near future.

## What Have We Learned From Birth Cohorts and Other Epidemiological or Clinical Evidence?

Alterations within the gut microenvironment are linked to AAS in children (38). Several studies suggest a negative correlation between farming lifestyle during early life and the risk of AAS (39, 40) while others identify a specific role for farm-milk consumption (41). Since “*correlation does not imply causation*,” evidence on the underlying immune mechanisms is needed.

### T Regulatory Populations: Tricks or Treat?

Recent findings show that the neonatal gut microbiota is different among children concerning AAS risk, and prove a different propensity to induce specific T cell responses. The analysis of gut microbiota of the WHEAL cohort, in which neonates and infants are clustered for AAS risk, demonstrates that neonates at higher risk exhibit a delayed diversification of the gut microbiota and a relative difference in the composition with fewer *Lactobacillus, Bifidobacterium, Akkermisia* and *Faecalicaterium* and more *Candida spp* (42). Sterile fecal water from these subjects impairs *in vitro* Tregs differentiation, while a reduction of CD4^+^CD25^+^FoxP3^+^ Tregs and an increase in IL-4^+^CD4^+^ T cells is observed (42).

The analysis of peripheral blood cells in PASTURE and EFRAIM children cohorts display an increase in CD4^+^CD25^+^FoxP3^+^ Tregs at 4.5 years (43) and a subsequent reduction at 6 years of age (44). Farm-milk is associated with a protective effect on AAS at 4.5 years of age, and this protection is partially dependent on Tregs. Beyond the number, the demethylation pattern of the FoxP3 promoter increases in farm-milk children but not in children with farm-exposure only (43). However, the longitudinal assessment of this cohort at 6 years of age finds no differences in functional assays nor Tregs frequencies among high or low farm milk intake groups, but an increased expansion of Tregs after LPS stimulation was demonstrated in children affected by AAS (44). Together, these studies support the hypothesis that the number of Tregs in peripheral blood is not a hallmark of tolerogenic responses.

### The Impact of Antibiotics: A Difficult Assessment in “Real-Life”

Some studies address the role of early antimicrobials administration and AAS. Early exposure to beta-lactams or macrolides has a differential impact on the development of the microbial community in children (45). Infants who are exposed to antibiotics early in life are more prone to develop AAS in a variety of cohort studies, but it is difficult to extract some conclusions on the shaping action on T cell response. Since oral intake is one of the most common routes of administration, it is rational to hypothesize a connection between antibiotics induced gut-dysbiosis and a Th2 polarized response. However, to our knowledge, no clustered analysis is available to date and evidence that link antibiotics induced gut dysbiosis and T cell response in AAS are currently lacking in these clinical scenarios.

### Effects of Preventive Probiotics Administration on T-Cell in Children

Interestingly, *Lactobacillus spp* is a constituent of genitourinary microflora and is an essential element of the microbiota in vaginally delivered-infants (46). Several shreds of evidence remark the role of delivery mode in determining the AAS risk in children, but other studies find no differences between elective cesarean and vaginal delivery (47). *Lactobacillus* is one of the strains depleted in high-risk toddlers; thus, it is hypothesized that supplementation could prevent the onset of AAS in children through a persistent modification of the microbiota. Since the therapeutic window is probably confined within the first year of life, the longitudinal comparison of stool samples collected from infants at high risk of AAS, treated, respectively, with *Lactobacillus rhamnosus GG* or placebo for 6 months was performed (48). While children at high risk show a distinct meconium microbiota and an impaired gut diversification, the treatment with *Lactobacillus rhamnosus* is able to restore this alteration at 6 months of life, but this effect is lost at 1 year of age (48). Moreover, sterile fecal water from infants at high risk treated with *Lactobacillus rhamnosus* at 6 months, but not at 12 months, promotes CD4^+^CD25^+^FoxP3^+^ T regs expansion and IL-10 production in DC/T-cell assays (48). Therefore, *Lactobacillus rhamnosus GG* could promote a tolerogenic environment, but it does not persist. Of note, a randomized, double-blind controlled trial of *Lactobacillus rhamnosus* supplementation during the first six months of life failed to show any significant difference in AAS at 5 years of age (49). Significant limitations of these studies include the intrinsic difficulty in controlling confounding factors and the heterogeneous definitions of atopy risk and AAS. A deeper immunological characterization in infants treated with probiotics in relation to the risk of AAS is needed.

## Conclusions

The complex interactions between the gut microbiota and the T-cell response in AAS are only partially uncovered. Further pathways should be outlined such as the relationship with lungs resident memory T cells (50), the induction of differential response in effector or central memory Th2 (51) or if an immune shift similar to those observed during immunotherapy (52) could be achieved by microbiome manipulation. Clinical trials are difficult and sometimes tainted by several confounding factors and rarely emphasize aspects related to the T cell responses. Moreover, when perinatal interventions are considered, the result should be clustered into pre, post, and combinatory ones in order to understand the priming effect of the delivery mode (49). The clarification of the mechanisms beyond the gut-lung axis strongly encourages further efforts to explore the potential therapeutic roles of microbiota-based primary prevention of AAS during early infancy.

## Author Contributions

All authors listed have made a substantial, direct and intellectual contribution to the work, and approved it for publication.

## Conflict of Interest

The authors declare that the research was conducted in the absence of any commercial or financial relationships that could be construed as a potential conflict of interest.
